# Autoimmune Hepatitis: Serum Autoantibodies in Clinical Practice

**DOI:** 10.1007/s12016-021-08888-9

**Published:** 2021-09-07

**Authors:** Benedetta Terziroli Beretta-Piccoli, Giorgina Mieli-Vergani, Diego Vergani

**Affiliations:** 1 Epatocentro Ticino & Facoltà Di Scienze Biomediche, Università Della Svizzera Italiana, Lugano, Switzerland; 2 Institute for Research in Biomedicine, Bellinzona, Switzerland; 3 King’s College London Faculty of Life Sciences &, Medicine At King’s College Hospital, London, UK; 4 Paediatric Liver, GI and Nutrition Centre, MowatLabs, King’s College Hospital, London, UK; 5 Institute of Liver Studies, MowatLabs, King’s College Hospital, London, UK

**Keywords:** Autoimmune hepatitis, Autoantibodies, Indirect immunofluorescence, Automated assays

## Abstract

Circulating autoantibodies are a key diagnostic tool in autoimmune hepatitis (AIH), being positive in 95% of the cases if tested according to dedicated guidelines issued by the International Autoimmune Hepatitis Group. They also allow the distinction between type 1 AIH, characterized by positive anti-nuclear and/or anti-smooth muscle antibody, and type 2 AIH, characterized by positive anti-liver kidney microsomal type 1 and/or anti-liver cytosol type 1 antibody. Anti-soluble liver antigen is the only AIH-specific autoantibody, and is found in 20–30% of both type 1 and type 2 AIH. Anti-neutrophil cytoplasmic antibody is frequently positive in type 1 AIH, being associated also with inflammatory bowel disease and with primary/autoimmune sclerosing cholangitis. The reference method for autoantibody testing remains indirect immunofluorescence on triple tissue (rodent liver, kidney and stomach), allowing both the detection of the majority of liver-relevant reactivities, including those autoantibodies whose molecular target antigens are unknown. Of note, the current knowledge of the clinical significance of autoantibodies relies on studies based on this technique. However, immunofluorescence requires trained laboratory personnel, is observer-dependent, and lacks standardization, leading to ongoing attempts at replacing this method with automated assays, the sensitivity, and specificity of which, however, require further studies before they can be used as a reliable alternative to immunofluorescence; currently, they may be used as complementary to immunofluorescence.

## Introduction

Autoimmune hepatitis (AIH) is a chronic inflammatory disorder characterized by loss of tolerance towards hepatic autoantigens, leading to an autoimmune attack to the liver [1]. Clinical features of the disease include female preponderance, elevated serum immunoglobulin G (IgG) levels, positive circulating autoantibodies, interface hepatitis at liver histology, and a swift response to corticosteroid treatment [2]. AIH affects all ages and races, and is subdivided into type 1 (AIH-1) and type 2 AIH (AIH-2): AIH-1 is by far more common and affects both children and adults, whereas AIH-2 is mainly a paediatric disease [3]. AIH-1 is characterized by positive anti-nuclear antibody (ANA) and/or anti-smooth muscle antibody (SMA), whereas AIH-2 is characterized by positive anti-liver kidney microsomal antibody type 1 (LMK1) and/or anti-liver cytosol type 1 (LC1) antibody [4, 5]. AIH presentation is variable: it can present acutely with symptoms resembling those of viral hepatitis, e.g. malaise, nausea/vomiting, anorexia, joint and abdominal pain, accompanied by jaundice, dark urine, and pale stools; with fulminant hepatic failure and encephalopathy; insidiously, with non–specific symptoms (progressive fatigue, amenorrhea, headache, anorexia, joint and abdominal pain, diarrhoea, weight loss), lasting from 6 months to a few years before diagnosis; with established chronic liver disease and complications of cirrhosis and portal hypertension (hematemesis from oesophageal/gastric varices, bleeding diathesis, splenomegaly), without a previous history of jaundice or liver disease; and at times is diagnosed after an incidental finding of abnormal transaminase levels, without hepatic symptoms or signs. AIH therefore should be excluded in all patients for whom a clear alternative diagnosis is not reached [1, 2].

While untreated AIH has a poor prognosis, with a 56% mortality rate during a follow-up of 30–72 months, up to 90% of patients respond well to immunosuppressive treatment and have excellent long-term outcomes [6–9]: therefore, timely diagnosis and adequate treatment initiation are key to prevent disease progression. Circulating autoantibodies represent an essential diagnostic tool in clinical practice, being positive in up to 95% of AIH patients if tested according to dedicated guidelines [10]. Therefore, physician’s awareness of the clinical significance of autoimmune liver serology is a prerequisite for properly requesting autoantibody testing and for interpreting laboratory results. On the other hand, the clinical laboratory needs to adhere to established guidelines on autoimmune liver serology testing methods, including reporting all observed specificities to the clinician. This article offers a comprehensive overview on the current methods of detection and on the clinical significance of autoantibodies in AIH, as well as on future perspectives to improve their clinical utility.

## Methods of Detection

The reference method to test liver-related autoantibodies is still indirect immunofluorescence (IIF) on triple rodent tissue, i.e. liver, kidney, and stomach [11], on which the current knowledge of the clinical significance of autoimmune liver serology is based. This technique allows the simultaneous detection of the main liver-related autoantibodies, including ANA, SMA, anti-LKM1, anti-LC1, and the anti-mitochondrial antibody (AMA), which is the serological hallmark of primary biliary cholangitis (PBC) [12], but can occasionally be present in AIH [13]. A second major advantage of IIF is its capacity to detect autoantibodies whose target antigens are still unknown. However, it requires trained laboratory personnel, is observer-dependent, and is poorly standardized. Moreover, the quality of the substrates differs among laboratories/manufacturers and over time, the studies investigating clinical associations dating back to the 1970s and 1980s, when fresh rodent tissues were used in contrast to currently fixed and commercially available tissue slide substrates. Comparative studies are lacking. Therefore, attempts to replace IIF on triple rodent tissues with immunochemical techniques or with IIF on HEp-2 cells, a widely used cell line derived from a human laryngeal carcinoma, are ongoing, but need validation [14]. Solid-phase assays have been established and are constantly ameliorated for autoantibodies whose target antigens have been identified, i.e. anti-LKM1, anti-LC1, AMA, and to some extent, ANA and SMA.

According to the recommendations issued in 2004 by the Committee for Autoimmune Serology of the International Autoimmune Hepatitis Group (IAIHG), diluted patient serum is incubated with the tissue substrates, allowing tissue binding of any autoantibody contained in the serum targeting antigens present in the substrates [11]. After washing to remove unbound antibodies, a second, fluorochrome labelled anti-human antibody is added, and, after re-washing, the substrates are examined by ultraviolet microscopy. Characteristic IIF staining patterns are given by positive sera, which should be titrated to extinction. Anti-nuclear reactivities should be further characterized on HEp2 cells, which, thanks to their large nuclei, allow detection of the nuclear IIF patterns, which are of crucial clinical importance in the setting of autoimmune liver diseases (see below).

The conventional starting serum dilution is 1:10. In adults, the positivity cut-off is 1:40, whereas in children and adolescents titres from 1:20 for ANA and SMA and from 1:10 for anti-LKM1 and for anti-LC1 are considered positive, since autoantibodies are rare in healthy subjects of these age groups [11].

Molecular assays are usually based on purified or recombinant antigens attached to a solid phase, to which diluted patient serum is added: if the corresponding antibody is present in the test serum, it will bind to the antigen-coated solid phase and is detected in a subsequent step by adding anti-human antibodies labelled with a chemiluminescent or fluorescent agent, or with an enzyme- or radio-label.

Of importance, anti-soluble liver antigen antibody (anti-SLA), deemed to be the only AIH-specific reactivity, is undetectable by standard IIF, while it can be detected by solid phase assays, which should be part of the diagnostic work-up of every patient with acute or chronic liver disease [10].

## Anti-Nuclear Antibody

Historically, ANA is the first autoantibody that has been associated to AIH, therefore suggesting an autoimmune origin of the disease, at that time named “chronic hypergammaglobulinemic hepatitis” [15].

**Fig. 1:**
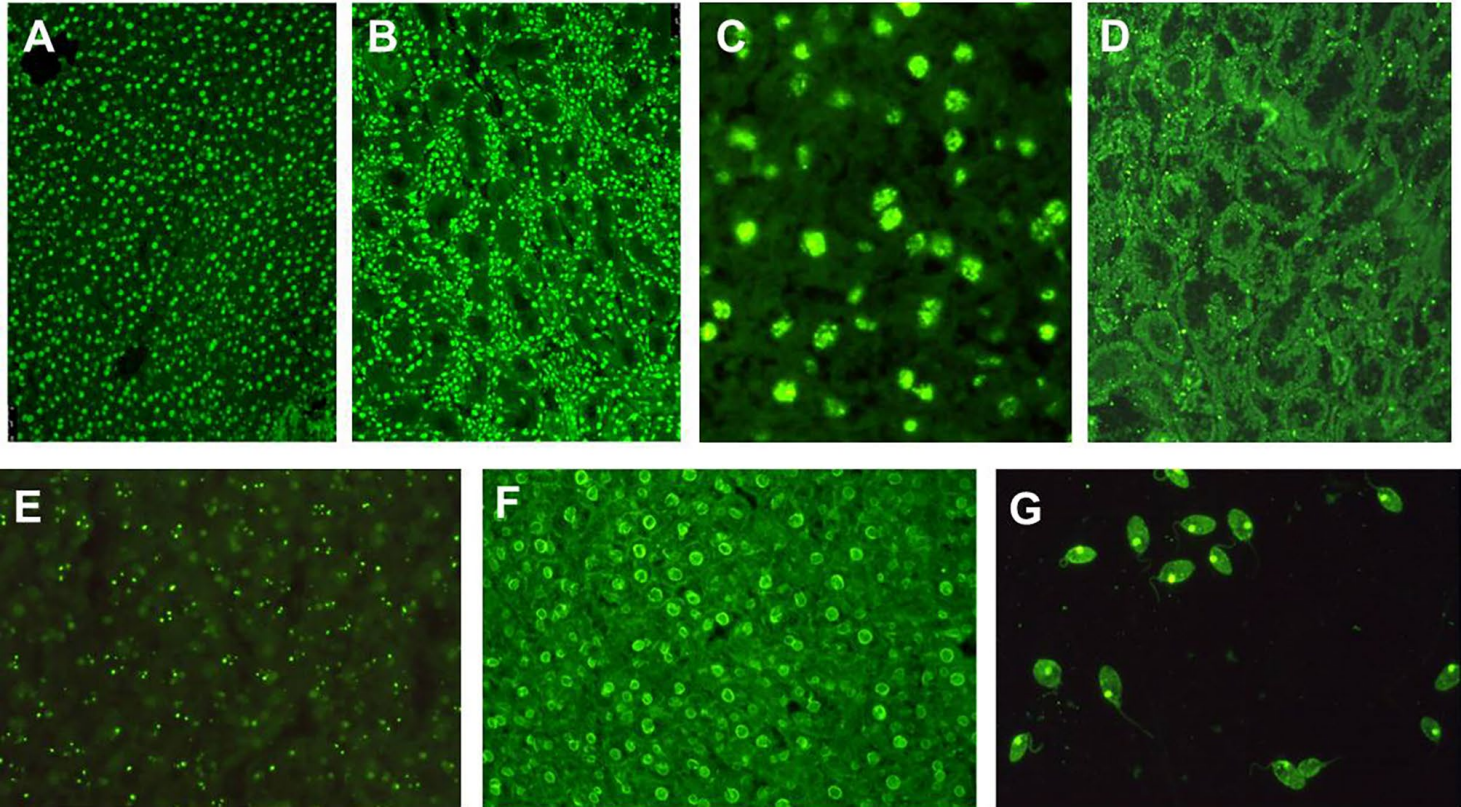
Anti-nuclear antibodies (ANA) detected by indirect immunofluorescence. **A** ANA with homogeneous pattern on rodent liver. **B** ANA with homogeneous pattern on rodent kidney. **C** ANA with speckled pattern on rodent liver, visualized at higher magnification. Anti-nucleolar antibody on rodent kidney **D** and liver **E**. **F** Peripheral or rim-like ANA on rodent liver. **G** Anti double-stranded DNA staining the kinetoplast of the flagellate parasite *Crithidia luciliae*. The patterns most commonly found in autoimmune hepatitis type 1 are **A** and **B**, followed by **C**, and much less frequently **D**, **E**, and **F**. Detection of anti-double stranded DNA on *Crithidia luciliae* has high specificity but low sensitivity

**Fig. 2:**
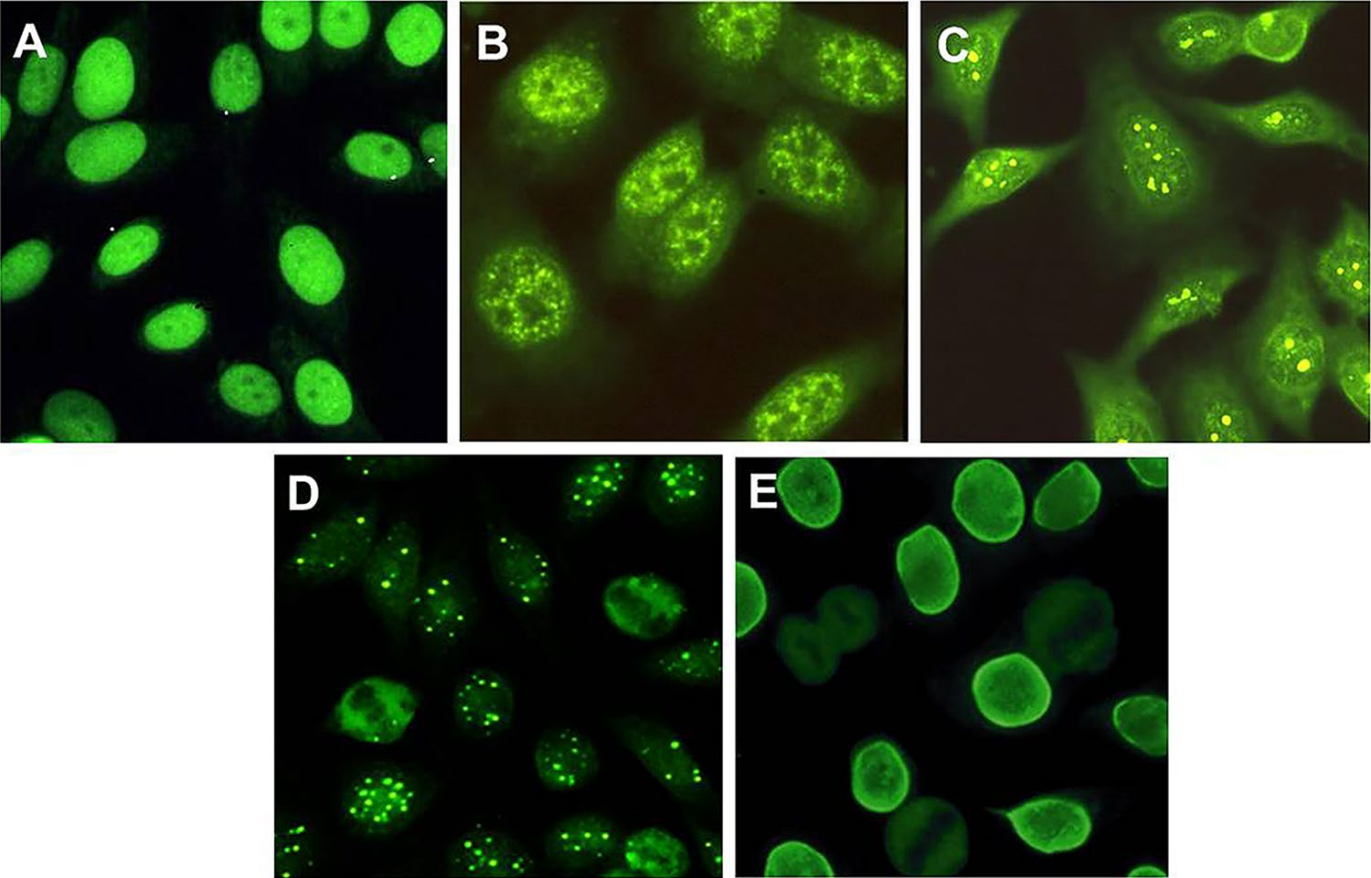
Anti-nuclear antibodies (ANA) detected on HEp2 cells by indirect immunofluorescence. The large nuclei of this laryngeal tumour cell line allow clear visualization of the ANA patterns. **A** Homogeneous. **B** Speckled. **C** Nucleolar. **D** Multiple nuclear dots. **E** Rim-like. **A** and **B**, and to a much lower frequency **C**, are found in autoimmune hepatitis type 1, **A** being by far the most common. **D** and **E** are patterns frequently encountered in primary biliary cholangitis

ANA should be tested by IIF, since nuclear target antigens in AIH are unknown in at least one third of the patients, leading to false negative results with potentially severe clinical consequences if only molecular-based tests are used [10] (Fig. 1). According to the recommendations issued by the IAIHG, HEp2 cells should not be used to investigate ANA at a screening level, since this substrate leads to potential false positive results, as low-titre positivity can be found in health [16, 17]. Nevertheless, this recommendation, which relates to the clinical context of liver diseases, is nowadays rarely followed by clinical laboratories, and HEp2 cells are widely used for ANA testing [18–20]. Therefore, HEp2 cells are used as a screening substrate also in the context of liver diseases, with a suggested higher positivity cut-off ≥ 1:160 [14]. The clinical performance of this approach needs to be validated in further studies. As mentioned above, HEp2 cells should be used to characterize the nuclear staining pattern of positive sera [11] (Fig. 2). Some three quarters of AIH patients show a homogeneous nuclear staining pattern on HEp2 cells, the remainder displaying a speckled or nucleolar pattern [10] (Fig. 2). Reported nuclear target antigens in AIH are listed in Table 1; among them, it should be mentioned that a strong association between anti-Ro52 and anti-SLA has been reported: therefore, anti-SLA should be tested in anti-Ro52-positive patients with liver disease [21]. Neither nuclear IIF patterns nor target antigens have been associated with specific clinical phenotypes to date [22].

**Table 1:** Clinical significance of autoantibodies in autoimmune hepatitis

Autoantibody	Autoantigens	Methods of detection	Frequency in AIH	Presence in other liver diseases	Clinical significance	Comments
ANA	Single- and double-stranded DNA Nuclear chromatin Histones Centromeres Cyclin A Ribonucleoproteins Unknown in at least 30%	IIF Solid-phase assays	60–70%	Drug-induced liver injury Viral hepatitis B, C, E Wilson PSC ASC PBC Fatty liver disease	Defines AIH-1, associated with SMA in 50% of the cases	IIF: homogeneous pattern in 2/3; speckled or nucleolar in 1/3
Anti-SMA	Filamentous actin Desmin Vimentin Unknown in 20%	IIF Solid-phase assay for anti-actin	Up to 85%	Drug-induced liver injury Viral hepatitis B, C, E Wilson PSC ASC Fatty liver disease	Strongly favors AIH-1 diagnosis, particularly if combined with ANA and at high titers	Titer correlates with disease activity in juvenile AIH-1 VG/VGT IIF patterns typical of AIH-1
Anti-LKM1	Cytochrome P4502D6 (CYP2D6)	IIF Solid-phase assays	At least 70% in AIH-2	Chronic hepatitis C	Diagnostic of AIH-2 after exclusion of hepatitis C	Titer correlates with disease activity in AIH-2
Anti-LC1	Formiminotransferase cyclodeaminase (FTCD)	IIF Solid-phase assays	30% in AIH-2	Very rare in hepatitis C, often with anti-LKM1	Diagnostic of AIH-2 after exclusion of hepatitis C	May be the only autoantibody in AIH-2 Titer correlates with disease activity in AIH-2
Anti-SLA	O-phosphoseryl-tRNA(Sec) selenium transferase	Solid-phase assays	Up to 20–30% in AIH-1 and AIH-2	Very rare in hepatitis C ASC	Diagnostic of AIH	Associated with more aggressive disease
pANNA	Betatubulin isotype 5 High-mobility group non-histone chromosomal proteins HMG1 and HMG2 Histone H1 Lactoferrin Elastase Catalase Alpha-enolase Bactericidal/permeability-increasing protein Other unknown antigens	IIF	20–96% in AIH-1	ASC PSC	May be the only autoantibody in AIH-1 PSC/ASC and inflammatory bowel disease should be excluded in pANNA-positive cases	Associated with inflammatory bowel disease Absent in AIH-2

*ANA* anti-nuclear antibody, *IIF* indirect immunofluorescence, *AIH* autoimmune hepatitis, *PSC* primary sclerosing cholangitis, *ASC* autoimmune sclerosing cholangitis, *PBC* primary biliary cholangitis, *anti-SMA* anti-smooth muscle antibody, *V* vessel, *G* glomerulus, *T* tubulus, *anti-LKM1* anti-liver kidney microsomal antibody type 1, *anti-LC1* anti-liver cytosol type 1 antibody, *anti-SLA* anti-soluble liver antigen, *pANNA* perinuclear anti-neutrophil nuclear antibody

ANA, coupled with SMA, defines AIH-1, being positive in about two thirds of the patients, associated with SMA in half of the cases [1, 23]. Seronegative AIH is rare, > 95% of the cases being positive for ANA and/or SMA, provided that autoantibodies are tested according to recommended cut-offs [11]. The clinician should be aware that ANA lacks disease specificity, being detected also in liver diseases different from AIH-1, particularly viral hepatitis B, C, D, and E, drug-induced liver injury, Wilson disease, alcohol-induced liver disease, and non-alcoholic fatty liver disease [24–28]. A variety of extrahepatic organ-specific and systemic autoimmune diseases are also typically associated with ANA-positivity, such as lupus erythematosus, Hashimoto thyroiditis, systemic sclerosis, or celiac disease, which may coexist with AIH [22]. Moreover, ANA may be positive even in healthy individuals, with frequency and titres increasing with age [16, 17].

Two ANA nuclear staining patterns are of particular clinical importance in the context of liver disease, i.e. the rim-like (also referred to as membranous) and the multiple-nuclear dot patterns (Fig. 2D, E), which, in association with a cholestatic biochemical profile, are characteristic of PBC, being particularly helpful in the diagnosis of AMA-negative PBC [12]. The main target antigen of the latter reactivity is sp100, whereas the anti-rim like antibody targets mainly gp210; molecular tests are available using these antigens, which however are not the only targets of the rim-like and multiple nuclear dot ANA, since both the nuclear membrane and the nuclear bodies, stained by the anti-rim-like and the anti-multiple nuclear dots, respectively, are complex structures [10].

Clinical laboratories are increasingly reporting positive cytoplasmic and mitotic immunofluorescence reactivities on HEp2 cells, according to the recommendations by the Executive Committee of the International Consensus on Antinuclear Antibody Patterns (ICAP) [20]. However, some laboratories, using HEp2 cells as substrate, report cytoplasmic staining as ANA, generating confusion in the clinical setting. Only Jo-1, targeting histidyl-tRNA synthetase in myositis and antisynthetase syndrome, which gives both a nuclear and cytoplasmic granular staining, may be referred to as ANA [29, 30]. Thus, there is an open ongoing discussion among international experts on how to address this issue. In the context of AIH, the clinical significance of these patterns is unknown and urgently needs to be investigated. Moreover, reporting a positive cytoplasmic pattern as ANA positive may lead to an incorrect score within the IAIHG diagnostic systems [31, 32]. To add more complexity to this issue, AMA, which provides negative points in the revised AIH scoring system [31], gives a cytoplasmic IIF pattern on HEp2 cells. The new and semantically more appropriate suggested nomenclature of “anti-cell antibody” has not yet been widely accepted, due to the potential major impact of a nomenclature change on disease classification criteria, methodologic consensus, and reimbursement policies [33].

## Anti-Smooth Muscle and Anti-Actin Antibodies

**Fig. 3:**
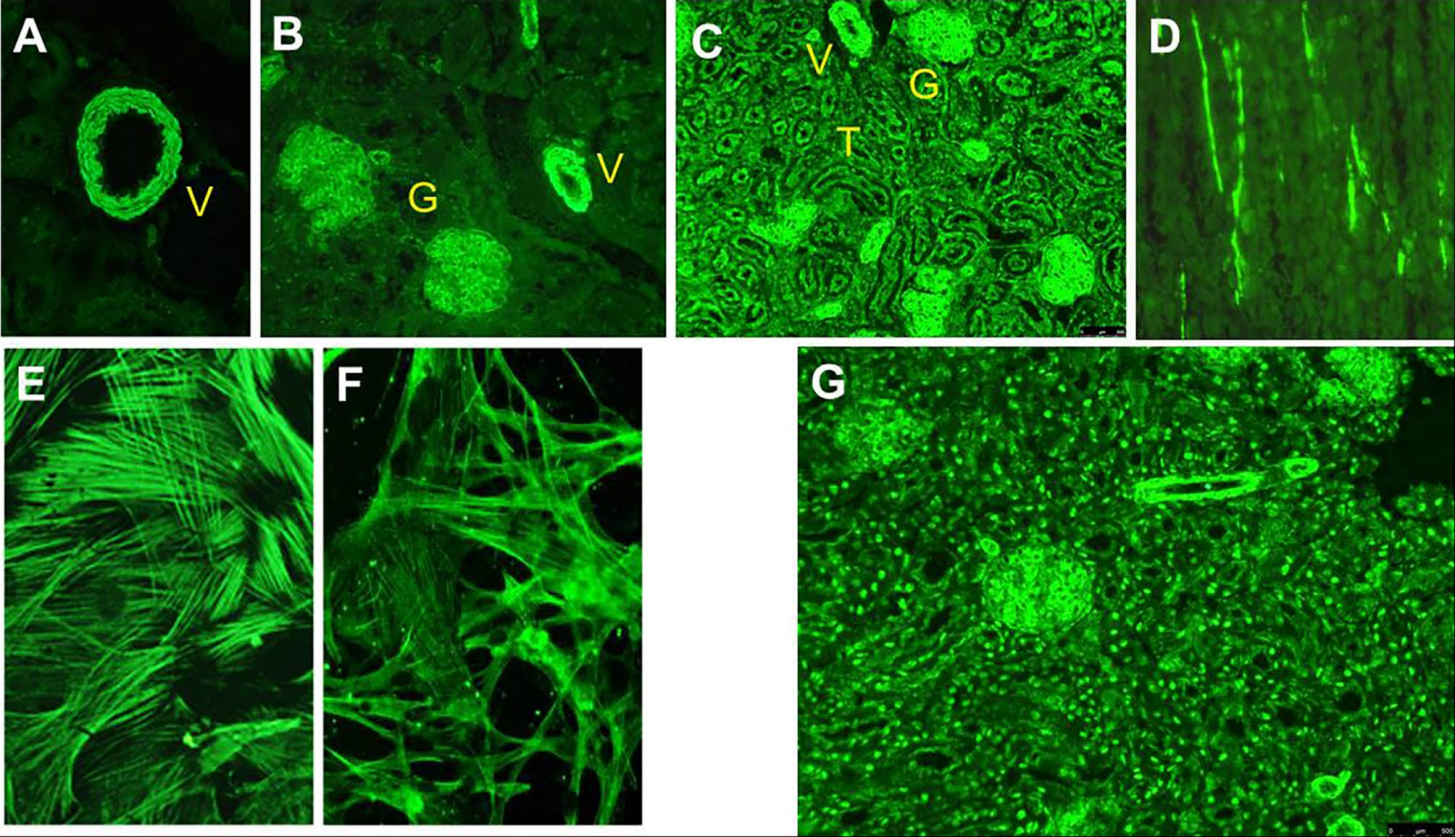
Anti-smooth muscle antibodies (SMA) detected by indirect immunofluorescence. **A**, **B**, and **C** Rodent kidney substrate; **A** vascular pattern (V); **B** vascular and glomerular pattern (VG); **C** vascular, glomerular, and tubular pattern (VGT). **D** Rodent stomach: staining of smooth muscle fibres between the gastric glands. **E** Human fibroblasts showing the so called “actin pattern” of SMA. **F** Vascular smooth muscle (VSM) 47 cells, also showing the “actin pattern.” **G** Rodent kidney showing the simultaneous presence of SMA with VG pattern and homogeneous ANA in a patient with autoimmune hepatitis type 1

The description of anti-SMA in 1965 contributed significantly to discriminate AIH from lupus erythematosus, this reactivity being absent in the latter condition [34]. On triple rodent tissue, anti-SMA stains the smooth muscle of the muscularis mucosa of the gastric wall, but also the smooth muscle of the mesangium of renal glomeruli and arterial walls [26] (Fig. 3). Bottazzo et al. recognized in 1976 three distinct IIF staining patterns on kidney tissue, namely vessel (V), vessel glomerular (VG), and vessel glomerular tubular (VGT) patterns [35] (Fig. 3). While the V pattern was detected also in serum of patients with viral infections, drug-induced liver injury, malignancies, fatty liver disease, primary sclerosing cholangitis (PSC) and PBC, the VG and VGT patterns were predominantly detected in serum of patients with AIH, at that time referred to as chronic active hepatitis [35, 36]. This association was validated in later studies [14, 37]. Therefore, clinical laboratory reports should include the IIF staining pattern of SMA-positive sera. The clinician, however, should be aware that some 20% of AIH SMA-positive patients lack the VG- or VGT-patterns, and, conversely, these patterns can be observed in liver diseases different from AIH, particularly viral hepatitis [10, 38]. The SMA titre is also of clinical significance, since higher titres have higher AIH-specificity [10, 27, 38]. If vascular smooth muscle cells (VSM) 47 cells, derived from rat embryonic thoracic aorta, are used as a substrate, sera giving the VGT pattern on kidney substrate give a typical cytoskeletal pattern (Fig. 3E). On a fibroblast substrate, these sera stain components of the cytoskeleton, giving the so called microfilament or F-actin pattern, suggesting that their antigenic target is an actin component [36, 37] (Fig. 3F). This observation led to the establishment of solid-phase assays using filamentous actin as an antigenic source, the results of which, however, are negative in some 20% of the VGT positive sera, indicating that additional, yet unknown, autoantigens are targeted by this reactivity, or, alternatively, that B cell epitopes are lost during protein purification [11, 36]. Therefore, anti-actin molecular assays should be used in a complementary manner to IIF on triple rodent tissue. On the other hand, anti-actin positivity is rarely found in SMA-negative AIH sera [39]. Moreover, low-titre anti-actin antibody can be found in patients with non-AIH liver diseases, particularly PBC and chronic hepatitis C, but are virtually absent in healthy controls [40, 41]. One study found an association of anti-actin antibody with more aggressive AIH [39].

In paediatrics, and possibly also in adults, SMA titres correlate with disease activity, and therefore are included in the definition of disease remission [42].

SMA, as mentioned before, defines AIH-1, being positive in 85% of the cases, associated with ANA in 50% [10]. The same autoimmune serological profile is shared with the paediatric condition referred to as autoimmune sclerosing cholangitis (ASC), which represents an overlap of AIH with an autoimmune attack to intrahepatic and/or extrahepatic bile ducts [9, 43, 44].

## Anti-Liver-Kidney Microsomal Antibody

**Fig. 4:**
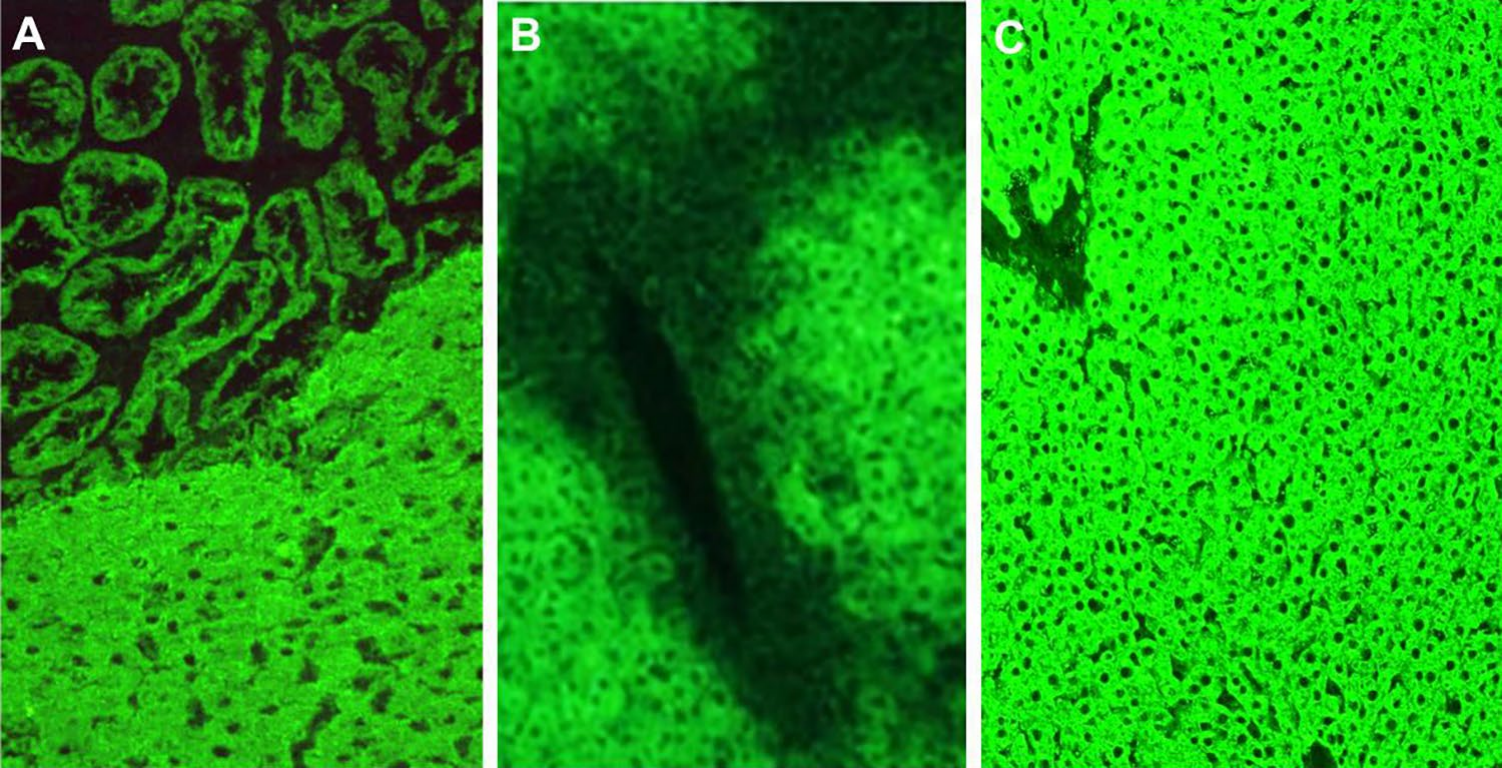
Anti-liver kidney microsomal type 1 (anti-LKM1) and anti-liver cytosol type 1 (anti-LC1) antibodies detected by indirect immunofluorescence on rodent tissue. **A** Anti-LKM1 on liver and kidney: strong staining of the cytoplasm of hepatocytes and of large renal tubuli. **B** Anti-LC1 on liver: the fluorescence typically declines towards the central vein. **C** Anti-LKM1 on liver, when **B** and **C** occur simultaneously; anti-LC1 is masked by the strong fluorescence of anti-LKM1 and should be investigated by molecular assays

Anti-LKM was discovered by Mario Rizzetto in 1973 in Deborah Doniach’s laboratory in London. He identified in the serum of a small fraction of a large group of patients with liver diseases an autoantibody that gave a bright staining pattern of the hepatocyte cytoplasm and of the proximal renal tubuli [45] (Fig. 4). Since the reactivity was abolished after incubation of the serum with a “microsomal fraction” obtained by ultracentrifugation of liver homogenate, the autoantibody was named anti-LKM [46]. The liver disease associated with anti-LKM was fully characterized by Alagille’s group in 1987 in Paris, who reported a cohort of 65 anti-LKM-positive patients, of whom 89% were female, and 56% were younger than 20 years, affected by an aggressive inflammatory liver disease, with a 14-year survival of only 51% despite treatment with prednisone and azathioprine [47]. This severe condition affecting mainly children and adolescents was named AIH-2; ANA, SMA, and AMA were reportedly absent in AIH-2 patients [47].

Anti-LKM1 is the serological hallmark of AIH-2, which is much rarer than AIH-1, accounting for 20–30% of paediatric and for up to 10% of adult AIH cases [2, 9, 48, 49].

Anti-LKM1 may be misdiagnosed by IIF, as AMA, particularly if the stomach substrate, spared by anti-LKM1, is not used [50]. The subtle differences between the two reactivities on liver and kidney substrates require a trained observer: on kidney tissue, anti-LKM1 stains the proximal larger tubules, whereas AMA stains more brightly the smaller, mitochondria-rich distal tubules; on liver tissue, the AMA staining pattern is much fainter as compared to the anti-LKM1 one [10, 22]. The identification of cytochrome P4502D6 (CYP2D6) as the target antigen of anti-LKM1 has allowed the establishment of reliable molecular-based assays, which allow to clarify doubtful cases [51–54]. Different immunodominant B-cell epitopes of this large protein have been subsequently identified, which are hierarchically recognized [55–57].

Anti-LKM1 titres correlate with disease severity in children with AIH-2 and should be used to monitor disease activity [42]. Furthermore, reappearance of anti-LKM1 antibody after liver transplantation for AIH-2 is associated with disease recurrence in the graft [58].

Anti-LKM1 is not entirely disease specific, being detected in up to 13% of patients with chronic hepatitis C infection [10, 38, 59]. The target epitopes of CYP2D6 of anti-LKM1 in AIH-2 and HCV show partial overlap, suggesting cross-reactivity between viral and self-epitopes [55, 60, 61]. Aminoacidic sequence homologies with CYP2D6 have been reported also for Cytomegalovirus and Herpes virus, leading to the “multiple hit hypothesis,” which states that exposure to self-mimicking sequences present in multiple common viruses may trigger AIH-2 in genetically predisposed subjects [62]. This pathogenetic mechanism is well illustrated by the case of a girl developing AIH-2 after exposure to several viruses, whose antigens have epitopes mimicking CYP2D6 [63].

Anti-LKM with slightly different IIF patterns and different clinical significance have been reported. De novo AIH, a disease entity indistinguishable from AIH arising in liver transplant recipients who underwent liver transplantation for diseases different from AIH, may be associated with an anti-LKM1 antibody giving an atypical IIF staining pattern, namely staining only kidney tissue, and referred to as atypical anti-LKM1 [64].

Anti-LKM2 has been identified in serum of patients with hepatitis induced by ticrynafen, also named tielinic acid, an uricosuric and anti-hypertensive drug withdrawn from the market in 1982 owing to its hepatotoxicity [46, 65]. This specificity, that targets CYP2C9, stains liver tissue in a inhomogeneous pattern, with more intense staining of the centrilobular hepatocytes; on kidney substrate, it stains preferentially the first and second portions of the proximal tubuli [66–68]. Anti-LKM3 has been detected in 13% of patients with chronic hepatitis delta by Crivelli et al. [69]. Its target antigen is family 1 uridine 5’-diphosphate glucuronosyltransferase (UGT-1). Anti-LKM3 needs to be tested on human or primate substrate, where it stains hepatocyte cytoplasm and proximal renal tubuli [70, 71]. Of note, anti-LKM3 has been found also in 19% of AIH-2 patients, rarely being the only serological marker [70].

Autoimmune polyendocrinopathy-candidiasis-ectodermal dystrophy (APECED) is a rare autosomal recessive disorder associated with mutations in the autoimmune regulator gene (AIRE), with some 20% of the patients affected also by an anti-LKM1-positive hepatitis [72]. Although the target antigens are CYP2A6 and CYP1A2, the IIF staining pattern on triple tissue is indistinguishable from AIH-2-associated anti-LKM1 [73].

## Anti-Liver Cytosol Type 1

Anti-LC1 antibody was first reported by Martini et al. in 1988, who identified it in serum of 21 patients with juvenile hepatitis, being the only serological marker in seven of them, and associated with anti-LKM1 in the remainder [74]. This early study already highlighted the disease-specificity of anti-LC1, which was absent in a large number of pathological and healthy controls [74]. The clinical features of AIH-2 anti-LC1-positive patients are indistinguishable from patients without this antibody [74]. On rodent liver substrate, this organ-specific reactivity stains brightly the hepatocyte cytoplasm, sparing the centrilobular zone [27] (Fig. 4). It can be masked by concomitant anti-LKM1 (Fig. 4), the two autoantibodies being often present in the same patient, making the commercial availability of reliable solid-phase assays very helpful [10]. Indeed, the target antigen of anti-LC1 has been identified as the formiminotransferase cyclodeaminase (FTCD), an intracellular enzyme catalyzing the conversion of histidine to glutamic acid [75, 76].

Anti-LC1, together with anti-LKM1, defines AIH-2. In about two-thirds of the cases, the two autoantibodies coexist, but in some patients, anti-LC1 is the only serological marker, making it essential for it to be investigated [23, 42, 74]. Anti-LC1 is very rarely detected in HCV patients, usually in association with anti-LKM1, and in patients with AIH-1 or ASC [77–79].

## Anti-Soluble Liver Antigen Antibody

Peter Berg in 1981 in Germany identified by complement fixation test an antibody in the serum of 20 patients with liver disease (18 female) reacting with the supernatant of rodent liver and pancreas homogenate, which he named anti-liver-pancreas (LP) antibody [80]. Response to prednisone treatment, ± azathioprine, was satisfactory in this small cohort of patients [80]. Some years later, the same group suggested that this specificity characterizes a distinct subgroup of AIH, namely type 3 AIH [81, 82]. Michael Manns in 1987 detected in the serum of 23 patients, mostly young women, an antibody recognizing an antigen contained in the supernatant of liver homogenate, which he called anti-soluble liver antigen (anti-SLA) [83]. Anti-SLA positive patients suffered from an aggressive form of hypergammaglobulinemic hepatitis, with good response to immunosuppressive treatment [83]. Since this reactivity was the only serological marker in 6/23 patients, also Manns suggested that it defines a third AIH subtype [83]. This suggestion was subsequently not approved by the IAIHG, since the sera in Manns’ paper had been tested using an ANA cut-off positivity higher than that recommended by the international AIH community [10]. Indeed, if tested at the recommended cut-off of 1:40, AIH patients with isolated anti-SLA positivity are rare [11]. Several years after the reports by Berg and Manns, it was recognized that anti-LP and anti-SLA are the same antibody, which is nowadays referred to as anti-SLA [25]. Of importance, anti-SLA is undetectable by immunofluorescence [22].

Anti-SLA is the only AIH-specific autoantibody, with a reported disease specificity as high as 98.9%, and has therefore a high value in the simplified IAIHG scoring system [1, 32, 78]. However, it is detected in only 20–30% of patients using commercially available solid-phase assays [10]. If sensitive radioligand assays are used, up to two-thirds of AIH patients are anti-SLA positive [84]. Of interest, anti-SLA is seen in both AIH-1 and AIH-2, a feature that, coupled with its high disease specificity, suggests a key pathophysiological role of its antigenic target, i.e. the intracellular enzyme O-phosphoseryl-tRNA(Sec) selenium transferase (SEPSECS), required for selenoprotein biosynthesis, originally identified by Gelpí in 1992, and later confirmed by Wies and by Volkmann as the molecular target of anti-SLA [85–88]. The reason why anti-SLA has low sensitivity in AIH is probably that molecular-based assays used in clinical laboratories only detect antibodies reacting with linear epitopes since they contain prokaryotically expressed antigens. Assays using eukaryotically expressed SEPSECS are cumbersome and still not suitable for clinical use. Epitope mapping of anti-SLA, using prokaryotically expressed protein, identified SEPSECS 395–414 as the immunodominant B cell epitope, overlapping with one HLA DRB1*0301-restricted T cell epitope [89, 90].

Besides being highly specific for AIH, anti-SLA is associated with more aggressive disease, namely more severe histology, longer time to achieve disease remission, more frequent relapse and need for liver transplantation, and death [84]. A recent study confirmed that anti-SLA positive AIH patients more often require lifelong immunosuppression, need longer time to achieve disease remission, and can less frequently be weaned from corticosteroid treatment as compared to anti-SLA-negative patients [91].

## Anti-Neutrophil Cytoplasmic Antibody

**Fig. 5:**
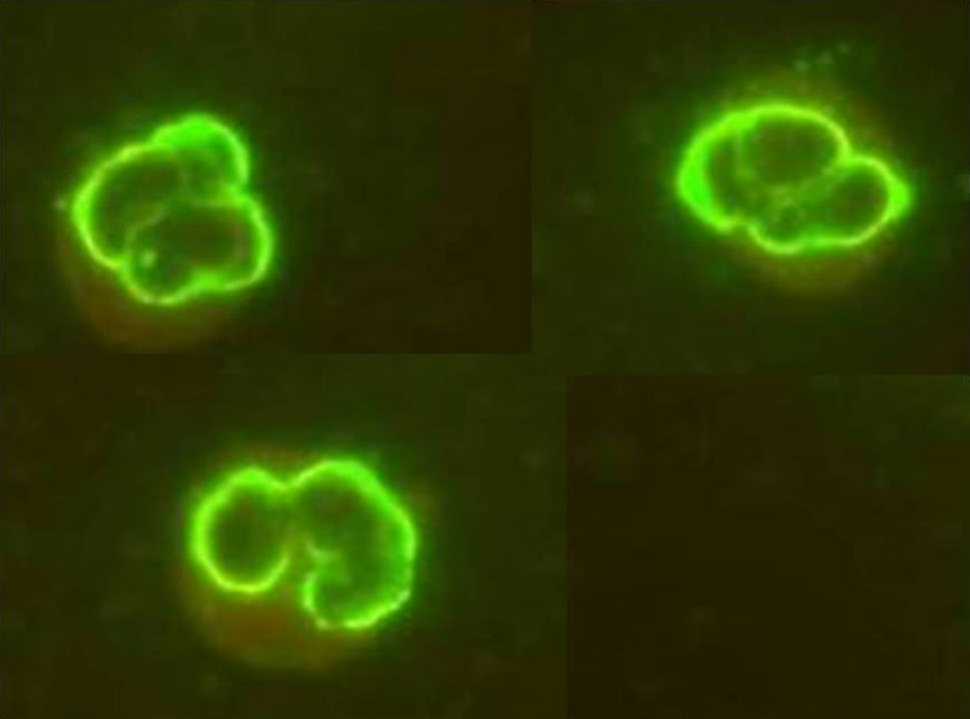
Anti-neutrophil cytoplasmic antibody with perinuclear pattern (pANCA), also known as perinuclear anti-neutrophil nuclear antibody (pANNA) detected by indirect immunofluorescence on human neutrophilic granulocytes. pANNA may be the only autoantibody present in autoimmune hepatitis type 1, though it is more frequently found in sclerosing cholangitis and inflammatory bowel disease (see text)

Anti-neutrophil cytoplasmic antibody (ANCA) has been first reported in the context of small- and medium-sized vessel vasculitis [92, 93]. It is detected by IIF on fixed human neutrophilic granulocytes, where different staining patterns are possible: cANCA display a diffuse cytoplasmic granular fluorescence, and pANCA display a perinuclear, often with nuclear extension, fluorescence [10]. While granulomatosis with polyangiitis is typically associated with cANCA, microscopic polyangiitis and eosinophilic granulomatosis are characterized by positive pANCA [94]. Both cANCA and pANCA target cytoplasmic proteins, the pANCA staining pattern being an artefact due to ethanol fixation of the neutrophils, which leads to migration of positively charged cytoplasmic proteins to the negatively charged nuclear cell membrane. Therefore, both ethanol and formalin-fixed neutrophils should be used to investigate ANCA. If the staining pattern is unaffected by ethanol fixation, the antibody is referred to as atypical pANCA, which targets components of the cell nuclear membrane, explaining its designation as perinuclear anti-neutrophil nuclear antibody (pANNA) [95] (Fig. 5). An alternative name for the same reactivity is nuclear anti-neutrophil antibody (NANA) [10]. The presence of ANA in the same serum may hinder pANCA detection owing to nuclear staining by ANA. As the main target antigen of cANCA is cytoplasmic protein leukocyte proteinase 3, and pANCA recognizes myeloperoxidase, molecular-based assays are commercially available for both [96]. This is not the case for pANNA, whose target antigens are only partially known, and in AIH-1 reportedly include betatubulin isotype 5, the high-mobility group non-histone chromosomal proteins HMG1 and HMG2, histone H1, lactoferrin, elastase, catalase, enolase, and bactericidal/permeability-increasing protein [96–98]. Interestingly, cross-reactivity of pANNA with betatubulin isotype 5 and the bacterial cell division protein FtsZ has been reported in PSC and AIH, suggesting a pathogenetic role of bacteria in these conditions [99].

Reported frequency of pANNA in AIH-1 ranges from 40 to 96%, being the only serological marker in a small subgroup of AIH-1 patients: therefore, it should be tested in patients with suspected AIH-1 and negative ANA, SMA, and anti-SLA [10, 100, 101]. cANCA is rare in AIH-1. In AIH-2, ANCA is virtually absent [10].

Besides AIH-1, ANCA is commonly found in PSC, with a reported frequency of up to 94% [102]. In children, ANCA frequency is higher in ASC as compared to AIH-1 [9]. Besides AIH, PSC, and ASC, pANNA is found in up to 80% of patients with inflammatory bowel disease [103]. Due to the association of AIH with sclerosing cholangitis and inflammatory bowel disease, we suggest that ANCA-positive AIH patients, particularly those with juvenile AIH, are screened for concomitant bile duct and bowel disease [43].

## Diagnostic Approach to the Patient with Suspected Autoimmune Hepatitis

While autoantibodies are an essential tool in the diagnostic jigsaw puzzle of AIH, they are not diagnostic on their own. Therefore, an essential knowledge of their testing methodologies and clinical significance is mandatory to use them correctly in clinical practice.

AIH should be considered in the differential diagnosis of every patient, of whatever age, presenting with liver disease of unknown origin, since AIH initial presentation is highly heterogeneous, ranging from asymptomatic to acute liver failure (see above) [1]. In addition to autoantibodies and serum IgG, liver histology is needed to confirm the diagnosis and to evaluate the severity of liver damage: interface hepatitis is the histological hallmark of AIH, but it is not pathognomonic, being found in other inflammatory liver diseases including viral hepatitis and Wilson disease. In acutely presenting AIH, and during relapses, panlobular hepatitis with centrilobular and bridging necrosis are often seen. In addition, liver histology is mandatory to diagnose PBC and PSC variant syndromes in adults, and ASC in children.

**Fig. 6:**
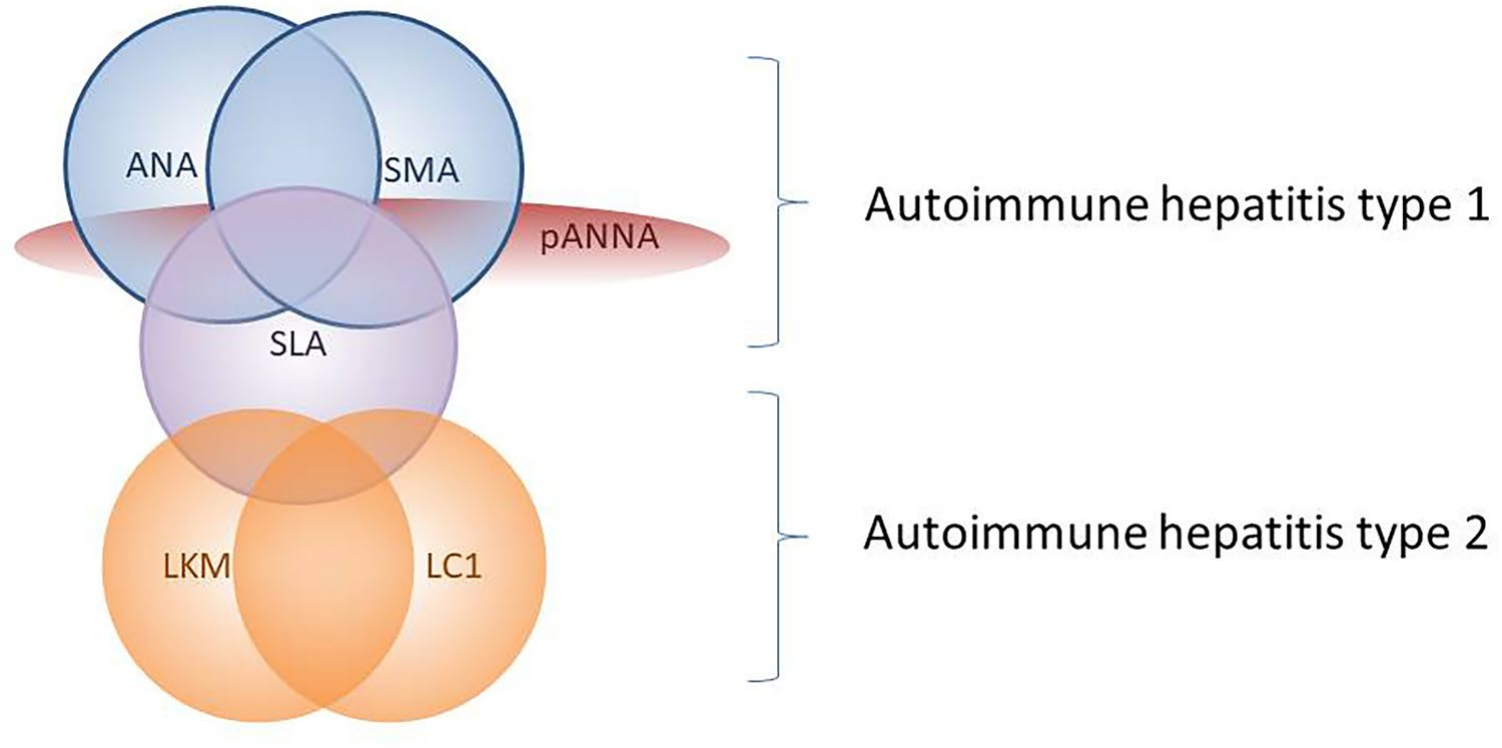
Summary of the clinical associations of autoantibodies in autoimmune hepatitis type 1 and type 2

Autoimmune serological workup (Fig. 6) should include IIF on triple rodent tissue and a solid-phase assay for anti-SLA [4]. This approach allows the detection of the majority of liver-related autoantibodies, i.e. ANA, SMA, anti-LKM1, anti-LC1, AMA, and anti-SLA. Combined positivity of ANA and SMA in the appropriate clinical context is highly suggestive of AIH-1, particularly if the SMA IIF pattern on kidney substrate is VG or VGT (Fig. 3). Anti-actin can be used as confirmatory test of SMA, or to exclude a rare anti-actin-positivity in the absence of SMA. Positive anti-LKM1 and/or anti-LC1 strongly suggest the diagnosis of AIH-2. AMA suggest PBC variant syndrome; however, AMA can transiently be positive in acute liver failure [104]. As mentioned above, isolated anti-SLA positivity is rare but possible in AIH-1. If this first set of autoantibodies does not show positive reactivities, ANCA should be tested using human neutrophils, since this autoantibody may be the only autoantibody present in a small subgroup of AIH-1 patients [10]. In juvenile AIH and ASC, autoantibodies should be used also to monitor disease progression, since titres correlate with disease activity [42]. A Brazilian study found that SMA titres correlate with disease activity also in adults with AIH-1, but further validation is needed before this can be included in the criteria of biochemical remission in the adult population [105].

## Reflections on Autoantibody Testing and Conclusion

Indirect immunofluorescence is an old technique requiring expertise and patience that may not be ideal for the modern laboratory. Yet, for the moment, it is here to stay.

Case number 5 of the Massachusetts General Hospital (MGH), published in 2009, is especially instructive and relevant to autoantibody testing [106]. It describes “A 47-Year-Old Woman with a Rash and Numbness and Pain in the Legs.” The patient had been suffering from pain and swelling of her legs, tender nodules, dry eyes and numbness, tingling, and colour changes in her fingers, for approximately 2 years. After physical examination and evaluation of her tests, Dr D Kroshinsky summed up: “This patient presents with a rash compatible with livedo reticularis, tender cutaneous nodules, atrophie blanche, mononeuritis multiplex, and peripheral-blood cytopenias. Serologic testing for rheumatologic diseases has been unrevealing.” The rheumatologist, Dr J H Stone, who examined the patient on admission at MGH, was asked for his possible diagnoses. The one he put on the top of the list was ANA negative systemic lupus erythematosus (SLE), noting that during the previous 2 years, ANA and anti-ds DNA were tested twice and resulted negative. He was puzzled since ANA is present in up to 99% of SLE patients and ordered new serologic tests. Indirect IIF on HEp2 cells showed an ANA positive at a titre of 1:1280. A repeat test was also positive. Could the patient have developed ANA over the 8 weeks before being seen at the Bostonian centre of excellence? The highly positive ANA sample on IIF was then sent to the commercial laboratory that had tested the patient’s sera previously. It was found to be negative. The technique unable to detect such a high titre ANA was flow cytometry–based solid-phase screening assay, in which coloured polystyrene beads coated with autoantigens are mixed with the patient’s serum, incubated with fluorescein conjugated antihuman IgG, and examined with the use of a dual-laser flow cytometer to detect both the colour of the bead and the amount of autoantibody coating the bead. The flow cytometry–based kit contains nine different-coloured beads coated with defined autoantigens: Ro, La, Sm, U1-RNP, Scl-70, Jo-1, Cenp-B, dsDNA, and histones. Importantly, a 10th bead is coated with material extracted from HEp-2 cell nuclei. This is a good example of how highly sophisticated tests, often advocated to replace old techniques, such as IIF, simply may not work.

Following this report and the fact that members of the American College of Rheumatology (ACR) were made aware of other inaccurate results for ANA testing, the College set up a Task Force to “evaluate the extent of the problem and to recommend solutions.” The Task Force reviewed the relevant literature and concluded that solid phase immunoassays may not be appropriate for replacing IIF as a screening test for the detection of ANA [19]. The key recommendations of the Task Force were that, first, IIF ANA test should remain the gold standard for ANA testing and, second, that hospital and commercial laboratories using bead-based multiplex platforms or other solid phase assays for detecting ANA must provide data that their assay has the same or improved sensitivity and specificity as IIF ANA.

More recently Pisetsky, in an Opinion published in Nature Reviews/Rheumatology [107], wonders whether: “Antinuclear antibody testing is misunderstood or misbegotten?” Misbegotten, as per the Cambridge dictionary, relates to something “badly or stupidly planned or designed.” Pisetsky reminds us that the nuclear antigens targeted by ANAs are usually present in the cell nucleus, though some may translocate to the cytoplasm, and that the HEp-2 cell line is used for ANA testing in IIF because this cell line displays a wide variety of antigens. HEp-2 cell testing for ANA is indeed recommended as gold standard by the ACR. Pisetsky, however, notes that “unfortunately, the gold standard does not have the brightness and lustre often ascribed to it” since “IIF can be subject to variability related to the assay kit used, conditions of cell fixation, cellular concentration of antigens and the specificity of the anti-IgG reagents.” Other issues raised are the initial dilution of the sample and the need for an expert observer. These limitations of the IIF technique have led to an interest in developing assays that might detect ANA more reliably, provide a higher throughput, in association to a decrease demand for experienced personnel and a higher cost efficiency. Pisetsky’s review of the available observer-independent assays, however, did not fare particularly well. ELISAs detect antibody of low avidity, while multiplex assays may provide results that require confirmation by IIF. Moreover, at variance with IIF, antigens bound to the inert support in the solid phase immunochemical tests tend to lose conformational epitopes, arguably the most relevant targets of disease specific, possibly pathogenic, autoantibodies.

In conclusion, to date, the least misbegotten test for the detection of ANA remains indirect IIF on tissue or cell lines, and this applies to the other autoantibodies relevant to autoimmune liver disease. The outstanding issue is standardization, both for IIF and immunochemical assays, and efforts in this direction are currently ongoing at international level. An associated corollary is the need of not losing the expertise in “reading immunofluorescence,” a prerogative belonging to a dwindling number of professionals, at least until artificial intelligence can do equally well or better than trained human eyes.
